# Revisiting the sclera as a target for glaucoma therapy

**DOI:** 10.1038/s41433-026-04615-w

**Published:** 2026-07-13

**Authors:** Seungsoo Rho, Young In Shin, Andrew Want, Julie Albon, James E. Morgan

**Affiliations:** 1 School of Optometry and Vision Sciences, Cardiff University, Cardiff, UK; 2 Department of Ophthalmology, CHA Bundang Medical Center, CHA University, Seongnam, Republic of Korea; 3 Department of Ophthalmology, Gachon University Gil Medical Center, Incheon, Republic of Korea; 4 Department of Ophthalmology, Gachon University College of Medicine, Incheon, Republic of Korea; 5 Vivat Scientia Bioimaging Laboratories, Cardiff University, Cardiff, UK; 6 University Hospital of Wales, Heath Park, Cardiff, Wales UK

**Keywords:** Optic nerve diseases, Prognostic markers, Vision disorders

## Abstract

The pursuit of improved intraocular pressure (IOP) control in glaucoma continues to drive innovation beyond conventional trabecular-based therapies. While minimally invasive glaucoma surgery (MIGS) has focused attention on enhancing conventional outflow, increasing evidence suggests that the sclera represents a biologically active and potentially modifiable interface influencing unconventional aqueous drainage and transscleral drug delivery. Traditionally regarded as a passive structural barrier, the sclera is now recognized as a spatially heterogeneous, mechanosensitive tissue with region-specific extracellular matrix composition and biomechanical properties. Emerging data demonstrate that pharmacologic agents such as prostaglandin analogues can remodel scleral extracellular matrix architecture, increasing molecular permeability and facilitating uveoscleral outflow. At the same time, advances in transscleral drug delivery including iontophoresis, ultrasound-assisted diffusion, biodegradable matrices, and extracellular vesicle–based platforms highlight the sclera’s dual role as both a hydraulic regulator and a therapeutic conduit. However, translational challenges including diffusion-dominated transport, episcleral clearance, fibrotic encapsulation, and dose variability have, to date, limited the widespread clinical adoption of transscleral strategies. In this review, we synthesize structural, biomechanical, pharmacologic, and translational evidence to propose a refined framework: the sclera as a dynamically remodeling hydraulic interface that may be selectively modulated to influence aqueous outflow and enable targeted posterior segment delivery. This integrative perspective expands the therapeutic paradigm for glaucoma beyond trabecular intervention alone.

## Introduction

Glaucoma is a leading cause of irreversible blindness worldwide, with its socioeconomic burden increasing as populations age [1]. The primary therapeutic goal is intraocular pressure (IOP) reduction, achieved via medications, laser, or surgery. However, treatment responses vary, and patient adherence, especially to topical therapies, remains suboptimal [2, 3].

The treatment of glaucoma has been transformed by the introduction of prostaglandin (PG) medications in the 1990s. Collectively, these remain the most commonly used topical medications for the reduction in IOP. The effect of PGs on IOP was opportune, coming from the observations that IOP was often reduced in conditions where PG production was high, for example, in uveitis [4]. Alm et al. identified the critical importance of uveoscleral drainage, which was previously thought to play a minor role in controlling the outflow of aqueous [5]. These studies clearly identified the potential for modification of scleral conductivity as a route for the control of IOP. Later work on changes in hydraulic conductivity confirmed that this changed with PG use, which focused attention on the role of scleral conductivity in regulating IOP as part of the uveoscleral outflow pathways [6]. More recently, scleral conductivity has been targeted as a potential route for intraocular delivery of topically applied medications, particularly for the treatment of retinal conditions [7]. These novel approaches have rekindled interest in the importance of modulating scleral conductivity for the regulation of aqueous outflow. In this review, we consider the potential roles of scleral modification in glaucoma management to facilitate the outflow of aqueous and highlight studies that may provide insight into potential progress needed for novel therapeutic options.

## Structure of sclera

The sclera is the most mechanically robust tissue of the eye, functioning as a critical barrier that protects delicate intraocular structures from mechanical stress. These stresses require the sclera to maintain both rigidity and resilience. Its thickness varies regionally—from approximately 0.3 mm beneath the recti muscles to up to 1.35 mm at the posterior pole—reflecting its functional adaptation to different stress environments. The scleral stroma (*substantia propria*) is the dominant layer conferring biomechanical strength [8]. It is composed of an intricate network of collagen fibrils, interspersed with proteoglycans and elastic fibres to form a fibrous matrix capable of withstanding both tensile and compressive forces. The superficial stroma includes the episclera, Tenon’s capsule, and extraocular muscle insertions, while the innermost layer, the *lamina fusca*, interfaces with the uveal tract. At the posterior pole, the sclera is continuous with the dura mater, with fibrillar collagen bundles oriented to reinforce the peripapillary region and mitigate mechanical vulnerability at the optic nerve head (ONH) [9, 10]. Anteriorly, the sclera blends with the peripheral cornea and interfaces with the trabecular meshwork and Schlemm’s canal. It also forms a continuum with the ciliary muscle and pars plana, supporting the unconventional (uveoscleral) outflow pathway for aqueous humor [11].

The integrity of scleral microstructure is essential for the maintenance of globe shape and optical function. X-ray diffraction studies have shown that collagen fibrillar orientation is regionally adapted to local stress and strain [12], with particularly refined alignment in the peripapillary optic nerve region to buffer mechanical load at the nerve exit [10]. Wide-angle X-ray scattering (WAXS) [13] and small-angle light scattering (SALS) [14] techniques have been used to describe the ultrastructure of the peripapillary sclera. These studies have highlighted the importance of water content in determining extracellular matrix (ECM) structure and biomechanical properties. Tissue hydration state, as well as molecular size/shape, surface charge and lipophilicity, is crucial to determining the permeability of the sclera to exogenous compounds [15,16].

**Fig. 1:**
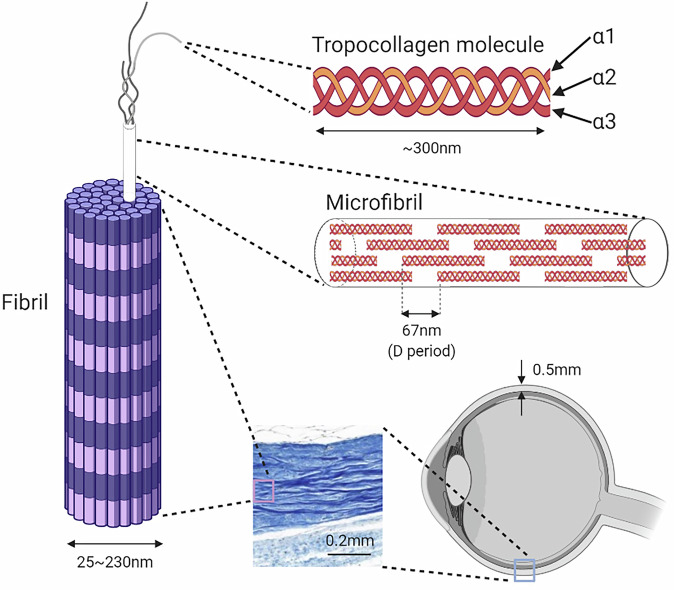
The hierarchical structure of the sclera collagen. Adjacent tropocollagen molecules with length of up to 300 nm are axially staggered by 67 nm (the D period) to form a microfibril. Collagen fibrils assemble into irregular bundles that eventually form the sclera of the eyeball. (histologic section of sclera; Masson-trichrome stain of a New Zealand White rabbit; unpublished data).

Studies at the nano-structural level have provided further insights into the importance of scleral fibre orientation (Fig. 1) [17, 18]. Transmission electron microscopy (TEM) of the outer scleral stroma has revealed a lamellar structure comprising collagen fibril bundles associated with proteoglycans. The latter were identified as fine filaments, representing the sulphated glycosaminoglycans component, positioned adjacent to the d and e bands of the D period of collagen fibrils [17, 18]. Scleral strength and resilience are conferred by the parallel collagen fibrils in the superficial layer and a denser, interwoven architecture in the deep stroma. Scattered elastic fibres are distributed between and within the collagen bundles throughout the stroma. In the innermost *lamina fusca*, smaller collagen bundles branch into the underlying choroidal stroma.

### Tissue Composition

The sclera comprises a dense connective tissue organized into a fibrous matrix of collagen and elastin, interwoven with proteoglycans and glycoproteins. Collagen types I, III, V, and VI are present, with type I predominating and type III constituting less than 5% [19]. Although elastic fibres account for only 0.6–2% of scleral dry weight, they serve a critical functional role, particularly in the deep perilimbal sclera, trabecular meshwork, and ONH. These fibres form an integrated and tensioned network that is thought to stabilize adjacent tissues and prevent the collapse of fluid-conducting pores during fluctuations in IOP [20, 21]. Despite their presumed importance in maintaining aqueous outflow dynamics, the functional significance of elastic fibres (with the exception of the trabecular meshwork), is yet to be determined [9]. Proteoglycans, consisting of a protein core and glycosaminoglycan (GAG) side chains such as chondroitin sulfate, dermatan sulfate, keratan sulfate, and heparan sulfate, play a key role in regulating collagen fibril organization and spacing [22]. The negatively charged sulfate residues on GAG chains attract water, creating a hydrated, incompressible gel that contributes to the load-bearing properties of the sclera and acts as a potential modulator of solute diffusion. The osmotic swelling pressure generated by GAGs has been well characterized in cartilaginous tissues, where it resists compressive forces. Although the relevance of this property in the sclera remains less clear, it is plausible that the same mechanism contributes to both biomechanical stability and selective permeability [9]. These features are of particular interest in the context of transscleral drug delivery, where tissue hydration, molecular sieving, and electrostatic interactions collectively influence the transport of therapeutic agents through the scleral matrix.

### Regional specialization of scleral extracellular matrix and cellular microarchitecture

Although the sclera is often described as a relatively homogeneous collagenous shell, substantial evidence demonstrates marked regional specialization in ECM composition and biomechanical behavior. Detailed structural analyses have shown that the posterior peripapillary sclera exhibits preferential circumferential collagen alignment surrounding the ONH, forming a biomechanical reinforcement ring adapted to regional mechanical load [9, 10, 23]. Wide-angle and small-angle scattering studies further confirm anisotropic collagen orientation patterns that differ significantly from those in equatorial and anterior regions [12, 14].

By contrast, the anterior sclera and perilimbal region demonstrate a more heterogeneous fibrillar organization and distinct proteoglycan composition. Age-related alterations in decorin, biglycan, and other glycosaminoglycans have been reported, influencing tissue hydration, osmotic swelling pressure, and mechanical compliance [17, 24, 25]. These biochemical differences may modify local hydraulic resistance and solute diffusion properties.

At the cellular level, scleral fibroblasts are mechanosensitive and actively participate in ECM remodeling. Experimental strain models have shown altered gene expression profiles in human scleral fibroblasts under mechanical load, including upregulation of matrix metalloproteinases and profibrotic signaling pathways [26, 27]. Moreover, experimental glaucoma models demonstrate dynamic collagen reorganization and increased cellular proliferation within the peripapillary sclera [23, 28]. Collectively, these findings indicate that the sclera represents a spatially heterogeneous and biologically responsive tissue rather than a uniform passive barrier.

### Anterior sclera as a hydraulic and cellular interface

While posterior scleral biomechanics have been extensively characterized [9, 10, 29], comparatively less attention has been directed toward the regional specialization of the anterior sclera. Anatomically, the anterior sclera forms the structural foundation of Schlemm’s canal, the scleral spur, and the collector channel system, positioning it as a critical interface in conventional aqueous outflow [30]. Microstructural studies have demonstrated that collagen fibril organization varies regionally across the globe, with preferential circumferential alignment in the peripapillary sclera and more heterogeneous fibrillar architecture in anterior regions [9, 12, 14].

Regional differences in ECM composition further contribute to functional heterogeneity. Variations in proteoglycan content and age-related alterations in decorin and biglycan have been reported in scleral tissue, influencing tissue hydration, osmotic swelling pressure, and biomechanical compliance [17, 24, 25]. These biochemical characteristics are likely to modulate hydraulic resistance and solute diffusion differently in anterior versus posterior sclera.

At the cellular level, scleral fibroblasts exhibit mechanosensitive behavior, altering gene expression and matrix remodeling pathways in response to mechanical strain [26, 27]. Experimental models of elevated IOP demonstrate increased scleral cellular activity and collagen reorganization, suggesting region-specific adaptive responses [23, 28]. Given its proximity to Schlemm’s canal and collector channels, localized modulation of anterior scleral biomechanics may influence aqueous percolation independently of trabecular meshwork function. Despite this anatomical and biological relevance, anterior scleral microarchitecture and cellular specialization remain relatively underexplored compared to posterior scleral biomechanics.

## Formation of outflow facility

### Conventional outflow associated with the sclera

**Fig. 2:**
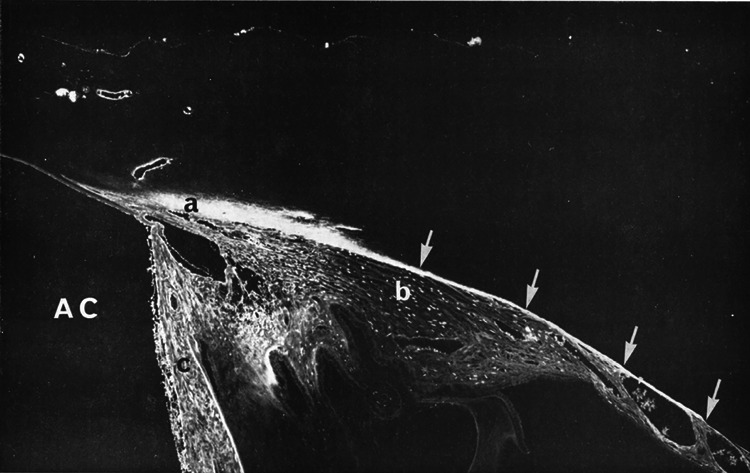
Fluorescein dextran in the unconventional outflow of a rabbit. Fluorescein dextran is noted in the conventional aqueous humor outflow pathway (**a**), comprising trabecular meshwork, aqueous plexus, and collector channels, and in the unconventional pathway of the ciliary body (**b**) and suprachoroidal space (arrows). Reproduced from Johnson M, McLaren JW, Overby DR. *Experimental Eye Research*. 2017;158:94-111, with permission from Elsevier.

Under normal physiological conditions, the bulk of aqueous humor exits the eye via the conventional outflow pathway, which includes the trabecular meshwork, Schlemm’s canal, and the episcleral venous system. The scleral sulcus, a shallow groove at the junction of the sclera and cornea, provides structural support for this pathway, helping it withstand physiological biomechanical stress [31]. Superficial scleral fibres are continuous with those of the cornea, while deeper fibres condense circumferentially to form the scleral spur—the anterior-most projection of the sclera. This rigid ring-like structure anchors the trabecular meshwork anteriorly and the longitudinal ciliary muscle posteriorly. Along with the corneal annulus, the scleral spur stabilizes the peripheral cornea and maintains the anatomical integrity of the conventional outflow system. Scanning electron microscopy has demonstrated this outflow continuum from the anterior chamber through the trabecular meshwork to Schlemm’s canal, and further into a deep intrascleral plexus, which communicates with more superficial intrascleral collector channels (Fig. 2) [32]. From there, aqueous humor drains into episcleral and conjunctival veins, completing its passage out of the eye [30].

### Unconventional outflow associated with the sclera: uveoscleral pathways

In addition to the conventional pathway, the sclera contributes significantly to unconventional aqueous humor drainage via the uveoscleral route. The aqueous humor may diffuse from the anterior chamber into the uveal tissue through interstitial spaces within the anterior uvea and the ciliary muscle, bypassing Schlemm’s canal entirely [33]. Tracer studies have demonstrated that once fluid enters the uveal tissue, it can exit the eye through several possible pathways [34–36]. One route is transscleral egress, where fluid passes through the sclera and episclera into the orbit, subsequently absorbed by orbital vasculature. Although the sclera lacks well-defined lymphatic vessels, its interfibrillar architecture may permit lymph-like drainage by acting as a porous matrix [37, 38]. From the episclera, lymph may drain into the subconjunctival space and eventually into the parotid or submandibular lymph nodes, depending on anatomical location [17, 39]. Another route involves osmotic absorption of aqueous humor by the choroid, with subsequent drainage through vortex veins embedded within the sclera. A third route may occur via lymphatic vessels in the ciliary body, where fluid exits through perivascular spaces and drains into the deep cervical lymph nodes via the jugular lymphatic trunks. Importantly, the relative contribution of uveoscleral versus trabecular outflow varies widely across species. In primates, the uveoscleral pathway can account for up to 50% of aqueous drainage, whereas in animals such as mice, dogs, and rabbits, this component is substantially lower [40–43].

The unconventional outflow pathway has been supported by multiple experimental approaches; however, the strength of evidence varies across the proposed mechanisms. Quantitative tracer studies in primates using radiolabeled or fluorescent macromolecules have consistently demonstrated that a substantial fraction of aqueous humor exits via the uveoscleral route, accounting for up to 40–50% of total outflow in young primate eyes [40]. These studies have provided the strongest evidence for interstitial flow through the ciliary muscle into the supraciliary and suprachoroidal spaces. Transscleral egress into orbital tissues has been suggested based on tracer distribution beyond the sclera and episclera, as well as histological observations of dye movement through scleral lamellae. However, direct quantification of bulk transscleral flow in vivo remains limited, and this pathway may represent a minor component relative to interstitial uveoscleral drainage. The role of lymphatic vessels in the ciliary body has gained attention following identification of lymphatic markers and photoacoustic imaging studies suggesting age-related decline in ocular lymphatic drainage [45]. Nevertheless, functional quantification of lymphatic contribution to steady-state aqueous humor outflow remains incomplete, and its relative contribution compared with classical uveoscleral interstitial flow is not fully established. Collectively, current evidence supports interstitial flow through the ciliary muscle and suprachoroidal space as the dominant unconventional pathway in primates, while transscleral and lymphatic mechanisms may contribute variably depending on species, age, and experimental conditions.

## Scleral contribution to IOP, ethnicity, and ageing

Numerous studies have demonstrated that the structural and mechanical properties of the sclera undergo significant changes in response to mechanical stress, aging, and glaucomatous damage. For instance, Pijanka et al. reported decreased collagen alignment in glaucomatous mouse eyes compared to contralateral controls. Similarly, SALS imaging by Jones et al. revealed greater collagen fibre alignment in the peripapillary sclera of healthy elderly eyes compared to both younger healthy eyes and glaucomatous aged eyes, suggesting a loss of biomechanical order associated with disease progression [10].

While much attention has focused on scleral structure, less is known about the cellular responses of the sclera to elevated IOP. Scleral fibroblasts are now recognized as active participants in mechanotransduction, capable of modifying ECM composition and transmitting mechanical signals via cytoskeletal networks. In vitro studies have shown that human scleral fibroblasts respond to mechanical strain by altering ECM gene expression. Moreover, high-magnitude or high-frequency mechanical loading has been shown to induce the differentiation of peripapillary scleral fibroblasts into contractile myofibroblasts, implicating IOP fluctuations as drivers of cellular remodeling. Consistent with these findings, experimental models have demonstrated up to a 6-fold increase in scleral cell proliferation following IOP elevation. Astrocytes in the lamina cribrosa (LC), which are glial fibrillary acidic protein (GFAP)-positive, also respond to elevated pressure and are thought to play a pivotal role in the initiation of glaucomatous optic neuropathy [44]. These glial responses are associated with multiple signaling cascades that can contribute to axonal injury and ECM remodeling. In parallel, LC cells that are GFAP-negative but fibroblast-like demonstrate pressure- and cytokine-induced ECM remodeling, particularly under mechanical stretch, transforming growth factor-β1 exposure, or hypoxic conditions [45, 46].

Genetic and epigenetic studies further support the sclera’s relevance in glaucoma pathogenesis. Park et al. reported racial differences in the epigenetic regulation of ECM-related genes—such as LOXL2, elastin, and fibrillin-1—in the sclera and LC, correlating with altered ECM expression between Caucasian and Korean populations [47]. Yan et al. also found that African American eyes exhibited fewer collagen fibres oriented circumferentially around the ONH compared to other racial groups, potentially contributing to differential biomechanical vulnerability [48]. Although histological measurements suggest that scleral thickness remains relatively constant with age, age-related declines in elastin content, decorin, and biglycan have been reported [17, 49]. Furthermore, the cross-sectional area of scleral collagen fibrils increases over time, likely due to the accumulation of advanced glycation end-products (AGEs), which contribute to tissue stiffening. Studies employing strip extensometry and inflation testing have confirmed that older scleral tissue exhibits increased stiffness in response to IOP, potentially altering fluid dynamics and susceptibility to optic nerve damage [50]. These findings suggest that the sclera is not a passive structural element but rather a dynamic, age- and race-sensitive tissue that may play a key role in glaucoma pathogenesis and progression.

### Clinical and pathological contexts in which the sclera is targeted

Beyond glaucoma, several ocular conditions provide insight into the therapeutic and pathological consequences of targeting scleral tissue. In inflammatory disorders such as scleritis and posterior scleritis, structural alterations of the scleral ECM are associated with edema, collagen degradation, and altered biomechanical properties, underscoring the sclera’s active biological role [51, 52]. In high myopia, progressive axial elongation is accompanied by scleral thinning, collagen remodeling, and changes in proteoglycan composition, illustrating how chronic biomechanical stress can induce region-specific ECM adaptation [53, 54].

Surgical interventions also directly modify scleral tissue. Procedures such as deep sclerectomy, canaloplasty, and implantation of glaucoma drainage devices involve partial scleral excision or remodeling, often altering local hydraulic resistance and fibrotic responses [55–57]. Similarly, cyclodestructive procedures target tissues adjacent to the sclera and may influence scleral permeability indirectly through ciliary body remodeling [58]. These clinical scenarios demonstrate that the sclera is not merely an inert barrier but a dynamic structure capable of remodeling in response to inflammation, mechanical stress, and surgical manipulation [9]. Examining these conditions provides valuable context for understanding both the therapeutic potential and biological limitations of scleral-targeted interventions in glaucoma.

## Therapeutic manipulation of scleral conductivity: clinical potential

The scleral stroma, a major outflow barrier, represents a logical target for IOP modulation. Mitomycin-C (MMC), when administered subconjunctivally as monotherapy, has demonstrated a significant IOP-lowering effect in both experimental models [59–61] and in human eyes following surgery [62, 63]. MMC primarily acts by inhibiting fibroblast proliferation and ECM deposition in subconjunctival tissues, thereby reducing postoperative scarring [64, 65]. Although this may indirectly influence local outflow resistance, direct quantification of intrinsic scleral hydraulic conductivity following MMC exposure has not been definitively established. However, early postoperative changes in IOP following MMC exposure may reflect transient effects on ciliary body function in addition to reduced fibrotic resistance. Because MMC readily diffuses across scleral tissue, dose adjustment is frequently necessary to avoid excessive suppression of aqueous humor production [66]. Surgical interventions such as deep sclerectomy—where a portion of scleral tissue adjacent to Schlemm’s canal is excised without full-thickness penetration—have demonstrated significant IOP reduction. Ultrasound biomicroscopy has confirmed that partial scleral removal enhances aqueous percolation, reinforcing the therapeutic relevance of scleral conductivity in non-penetrating filtration procedures [67]. Clinical observations from non-penetrating glaucoma surgery further support the concept that the extent and configuration of scleral tissue excision can influence postoperative aqueous egress and IOP control. In an intra-individual comparative study of ab externo canaloplasty, eyes undergoing classic deep lamellar dissection with deep scleral flap excision thereby creating a trabeculo-Descemet’s membrane (TDM) window and an intrascleral lake achieved slightly greater and more sustained IOP reduction than eyes treated with a flap-sparing vertical cut-down incision, despite both approaches being closed watertight and lacking bleb formation [68]. The authors suggested that additional outflow via percolation through the TDM window into the intrascleral lake, with potential access to episcleral venous and suprachoroidal routes, may contribute to this difference. Complementing these findings, a large retrospective series comparing conventional canaloplasty with a modified technique incorporating suprachoroidal drainage reported a significantly greater IOP reduction and a higher proportion of medication-free patients at 12 months in the modified group [69]. These data are consistent with the notion that altering the deep scleral dissection plane—and thereby reducing resistance to extra-trabecular egress—can augment pressure lowering beyond Schlemm’s canal/collector channel mechanisms alone.

PG analogues, particularly PGF2α derivatives, provide one of the clearest examples of pharmacologic modulation of scleral tissue. Beyond their established effects on the ciliary muscle, experimental evidence indicates that PGs act directly on sclera. In human scleral organ cultures, exposure to PGF2α, 17-phenyltrinor-PGF2α, or the active form of latanoprost (PhXA85) produced a dose- and time-dependent increase in transscleral permeability, quantified using a two-chamber Ussing apparatus. Notably, permeability increases were greatest for smaller macromolecules (10 kDa dextran), suggesting enlargement of intrascleral supramolecular pathways rather than nonspecific tissue disruption. These changes were accompanied by significant upregulation of matrix metalloproteinases, particularly MMP-2 and MMP-3, supporting the concept that extracellular matrix remodeling underlies the observed permeability shifts [70]. Importantly, these studies measured diffusion-based permeability coefficients rather than pressure-driven bulk hydraulic conductivity. Thus, while increased molecular permeability may facilitate interstitial fluid movement within the uveoscleral pathway, direct quantification of changes in intrinsic hydraulic conductivity under physiological pressure gradients remains limited. Consistent with these mechanistic observations, topical latanoprost administration has been shown to nearly double uveoscleral outflow in primate models without significantly affecting conventional trabecular drainage [71, 72]; these findings suggest that scleral extracellular matrix remodeling represents a pharmacologically accessible component of the unconventional outflow pathway. Interestingly, in experimental myopia models, latanoprost not only reduced intraocular pressure but also influenced scleral collagen organization and microstructural architecture, indicating that PG-mediated ECM modulation may extend beyond outflow regulation to broader biomechanical remodeling of scleral tissue [72].

## Sclera as a therapeutic target for glaucoma

Recent insights into scleral biomechanics suggest its role as both a structural support and a rate-limiting barrier for IOP control and drug delivery. With the growing use of biologic agents for retinal diseases, intraocular injections have become commonplace; however, they carry inherent risks such as endophthalmitis, retinal detachment, and patient burden due to repeated procedures. As a result, there is growing interest in non-invasive, transscleral delivery strategies that offer improved safety and patient compliance.

To understand the feasibility of transscleral therapeutic strategies, it is necessary to consider the fundamental determinants of scleral permeability as characterized in ocular pharmacokinetic studies. Extensive quantitative work in ocular pharmacokinetics has characterized the permeability properties of the sclera. Experimental studies have demonstrated that transscleral diffusion is primarily governed by molecular weight, hydrodynamic radius, surface charge, and tissue hydration state [15, 16]. Smaller, hydrophilic macromolecules exhibit higher diffusion coefficients, whereas permeability decreases exponentially with increasing molecular size [73]. Charge interactions between solutes and the negatively charged glycosaminoglycan matrix further influence transport kinetics. Although electrostatic attraction may facilitate diffusion of oppositely charged molecules, studies using dextran models have shown that net surface charge may affect molecular conformation and tissue interaction in complex ways rather than through simple ionic attraction alone [74, 75]. Importantly, transscleral transport appears to be predominantly diffusion-driven under physiological conditions, with limited contribution from bulk convective flow [73]. Tissue thickness, regional hydration gradients, and episcleral vascular clearance also significantly influence effective drug delivery to posterior segment structures. These findings suggest that while the sclera is permeable to a wide range of macromolecules, its permeability is neither uniform nor unrestricted, and successful therapeutic translation requires careful consideration of molecular design and delivery kinetics.

Efforts to modulate scleral hydraulic conductivity have largely focused on facilitating drug penetration to the posterior segment, rather than enhancing aqueous outflow. Nevertheless, the technologies developed for transscleral drug delivery provide a conceptual and practical foundation for targeting the sclera as a route for IOP reduction. For instance, iontophoresis has been shown to enhance scleral permeability by applying a low electrical current to drive charged molecules, such as dexamethasone, into intraocular tissues. Behar-Cohen and colleagues have demonstrated that the transscleral iontophoresis of dexamethasone achieved therapeutic effects comparable to systemic corticosteroid therapy in uveitic models [76]. Similarly, phonophoresis, which uses ultrasound waves to enhance scleral diffusion of non-charged compounds, has shown promise. Lamy et al. reported that 880 kHz ultrasound significantly increased the intraocular penetration of fluorescein through the sclera into the vitreous [77].

The sclera’s permeability is influenced by several physicochemical properties of the diffusing agent, including molecular weight, hydrophilicity, shape, and net surface charge [75]. Studies using porcine sclera—widely accepted as a surrogate for human scleral tissue—have demonstrated that smaller, hydrophilic, and negatively charged molecules diffuse more readily [75]. Proteoglycans in the scleral ECM carry negative charges, potentially facilitating the movement of oppositely charged solutes. However, Srikantha et al. showed that positively charged dextrans had higher diffusion coefficients than neutral or negatively charged forms [75], suggesting that surface charge may affect transscleral diffusion indirectly by altering molecular conformation or tissue interaction, rather than through direct electrostatic attraction alone [73]. These complexities emphasize the need for careful molecular design in developing drugs intended for transscleral delivery.

**Fig. 3:**
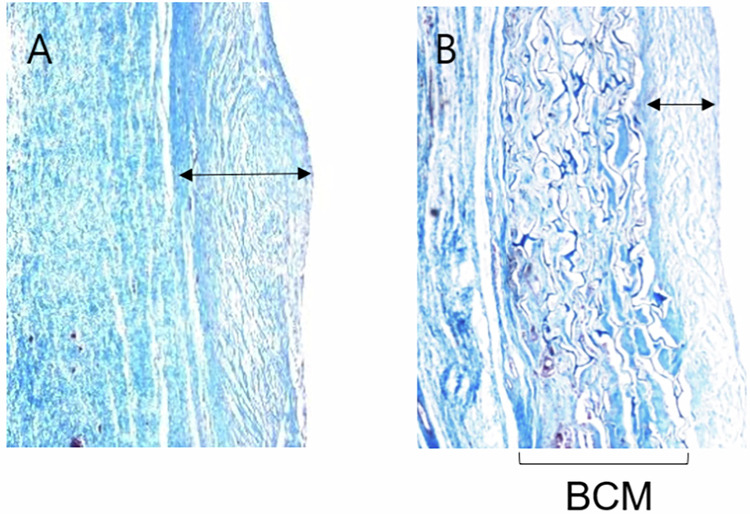
A biomechanical change on conjunctival fibrosis by a biodegradable collagen matrix (BCM). **A** The thickness of the fibrosis (*double-headed arrow*) over the Ahmed glaucoma valve (AGV) plate in the control group. **B** The thickness of the fibrosis (*double-headed arrow*) over the AGV in the BCM treated group. Note that the BCM treated group showed thinner fibrotic tissue 1 month after AGV implantation. (Masson-trichrome stain, New Zealand White rabbit).

Despite its potential, transscleral or subconjunctival drug delivery faces several limitations. One major obstacle is that fibrotic encapsulation can occur at the delivery site, which would impair sustained drug diffusion and limit repeatability. This is particularly relevant in glaucoma, where fibrous tissue formation is a major contributor to surgical failure [78, 79]. To address this, drug delivery systems incorporating biodegradable materials—such as poly(lactic-co-glycolic acid) (PLGA) or collagen matrices have been explored. Jessen et al. reported minimal fibrous reaction to PLGA-encapsulated latanoprost over three months in rabbits, although fibrosis increased over longer durations [80]. Similarly, biodegradable collagen matrices implanted with glaucoma drainage devices have shown reduced fibrosis and improved postoperative IOP control. In our own preclinical experiments, Ahmed glaucoma valves implanted with collagen matrix scaffolds exhibited significantly thinner fibrotic capsules compared to controls, suggesting a role for biomechanical modulation in optimizing drug delivery and surgical outcomes (Fig. 3) [81].

### Barriers to clinical translation of transscleral drug delivery

Despite these technological advances, transscleral drug delivery has not yet achieved widespread clinical adoption. Several biological and pharmacokinetic barriers may account for this translational gap. First, episcleral and conjunctival vascular clearance can rapidly remove drugs applied to the external scleral surface, reducing effective intraocular penetration [82, 83]. In addition, the high perfusion of the choroid creates a “vascular sink” effect, whereby compounds reaching the posterior segment may be cleared before achieving sustained therapeutic concentrations [74, 84]. Second, under physiological conditions, transscleral transport is predominantly diffusion-driven, with minimal bulk convective flow [15, 73]; this limits the ability to achieve high and reproducible intraocular drug concentrations, particularly for large biologic molecules [15, 75]. Variability in scleral thickness, hydration, and regional microarchitecture further complicates dose predictability across patients [85]. Third, fibrotic encapsulation and wound-healing responses following subconjunctival implantation or repeated drug exposure may progressively increase diffusion resistance [60, 61]. Fibrotic scarring is particularly relevant in glaucoma, where fibroproliferative responses are common after surgical manipulation [86]. Finally, regulatory challenges and limited long-term pharmacokinetic data have hindered the development of standardized, reproducible delivery platforms [74]. Together, these factors explain why, despite favorable permeability characteristics, transscleral delivery remains largely investigational rather than clinically established.

## A new target for outflow enhancement

**Fig. 4:**
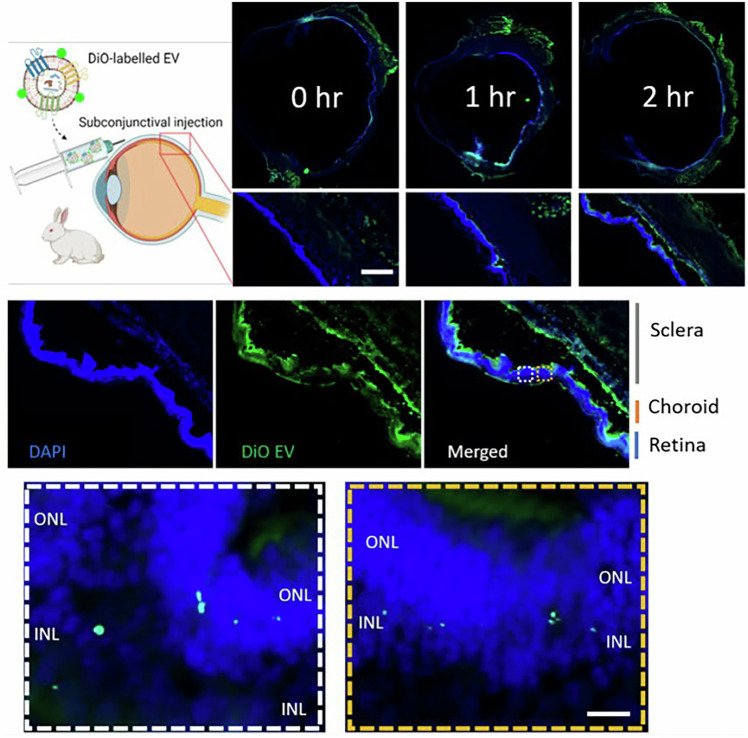
Retinal ganglion cell preservation following transscleral delivery of EV-encapsulated nicotinamide. Representative flat-mounted retinae stained with Brn3a demonstrate marked preservation of retinal ganglion cells (RGCs) in eyes treated with EV-encapsulated nicotinamide (EV-NAM) compared to untreated controls. Quantitative analysis confirms a significant increase in Brn3a⁺ RGC density in the EV-NAM group relative to both control and free nicotinamide (NAM) groups. These findings support the neuroprotective efficacy of transscleral EV-mediated delivery platforms in preserving RGCs. Reproduced with permission from Kim M, Kim JY, Rhim W-K, et al. *Acta Neuropathol Commun*. 2024;12:65. Licensed under CC BY 4.0 (https://creativecommons.org/licenses/by/4.0/).

**Fig. 5:**
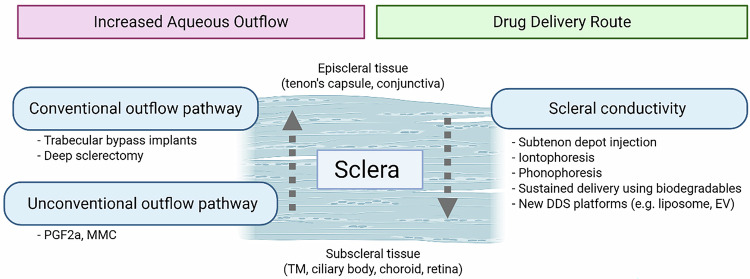
Scleral structure as a dual therapeutic target in glaucoma. The sclera contributes to both conventional and unconventional aqueous humor outflow, playing a key role in intraocular pressure regulation. In parallel, its extracellular matrix composition enables transscleral delivery of therapeutic agents, including sustained-release platforms and neuroprotective molecules such as EV-encapsulated nicotinamide. By modulating scleral conductivity, novel strategies can achieve both increased aqueous outflow and targeted drug delivery to posterior segment tissues.

Traditional glaucoma treatments have largely focused on enhancing trabecular or uveoscleral outflow and reducing aqueous humor production. However, increasing evidence supports the concept that the sclera is not merely a passive barrier but an active modulator of ocular fluid dynamics—and potentially a viable route for targeted therapy. The modulation of scleral permeability and biomechanics may allow for both enhanced outflow and efficient transscleral drug delivery. Recent work has expanded this paradigm by demonstrating that the sclera can be harnessed not only for pressure-lowering strategies but also for delivering neuroprotective therapies. A preclinical study provided compelling evidence that extracellular vesicle (EV)-encapsulated nicotinamide (NAM), delivered via a transscleral route, conferred robust neuroprotection to retinal ganglion cells (Fig. 4) [87]. This finding underscores the feasibility of transscleral delivery for not only modulating IOP but also to address the neurodegenerative component of glaucoma. These results open new possibilities for using the sclera as a conduit for sustained-release systems capable of reaching posterior segment structures, including the ONH. The mechanical and hydrophilic properties of the sclera—along with its proximity to key neurovascular tissues—make it a particularly attractive target for minimally invasive yet physiologically integrated therapies. In this expanded framework, scleral modulation is no longer limited to enhancing aqueous outflow but may serve as a bridge to a new class of neuroprotective and disease-modifying treatments. As drug delivery platforms become more sophisticated, integrating biomimetic carriers such as EVs with scleral-targeted routes could transform the management of glaucoma into a multi-dimensional therapeutic approach—simultaneously addressing IOP, tissue remodeling, and neuronal resilience. Taken together, these findings highlight the sclera’s dual therapeutic potential—as both a route to control IOP and a conduit for targeted drug delivery to posterior ocular tissues. A schematic summary of these complementary roles is illustrated in Fig. 5.

## Conclusions

While the concept of targeting the sclera for drug delivery or modulation of aqueous outflow is not new, recent advances in structural biology, biomechanics, and pharmacologic remodeling provide a more refined understanding of how and where such targeting may be feasible. Rather than viewing the sclera as a passive diffusion barrier, accumulating evidence supports its characterization as a spatially heterogeneous, mechanosensitive, and dynamically remodeling tissue. Regional specialization of ECM composition, differential collagen anisotropy between anterior and posterior sclera, and mechanosensitive fibroblast activity suggest that scleral properties are neither uniform nor static. These insights allow a shift from generalized “scleral targeting” to the more precise concept of selective modulation of anterior scleral hydraulic properties, potentially influencing unconventional outflow pathways without direct trabecular intervention. Furthermore, quantitative characterization of diffusion-based permeability, together with recognition of translational barriers such as episcleral clearance and fibrosis, highlights the need for integrated biomechanical and pharmacokinetic strategies. Future therapeutic approaches may therefore focus not only on enhancing drug penetration, but on controlling alterations in scleral microarchitecture to fine-tune interstitial fluid movement. In this framework, the sclera emerges not merely as a drug-access route, but as a modifiable biological interface bridging biomechanics, pharmacology, and aqueous humor dynamics. This integrative perspective may offer new opportunities for glaucoma therapy beyond conventional trabecular or suprachoroidal paradigms.
